# Perceptions and Experiences of an Attachment-Based Intervention for Parents Troubled by Intimate Partner Violence

**DOI:** 10.1007/s10615-016-0606-1

**Published:** 2016-09-01

**Authors:** Lana Kamal, Jennifer Strand, Göran Jutengren, Inga Tidefors

**Affiliations:** 1 Academy of Care, Working Life, and Social Welfare, University of Borås, 501 90 Borås, Sweden; 2 Department of Psychology, University of Gothenburg, Box 500, 405 30 Gothenburg, Sweden

**Keywords:** Domestic violence, Focus groups, Intervention, Parent–child attachment

## Abstract

It is known that intimate partner violence (IPV) negatively affects both parental capacity and children’s well-being, but few studies have focused on the experiences of those taking part in family interventions focused on IPV. In this study, 26 parents (16 mothers and 10 fathers) with a history of IPV participated in focus groups concerning their attachment-based group intervention experience in the program Parenting and Violence. The transcripts, subjected to thematic analysis, showed that participants experienced the intervention as supportive and confirming of their role as parents. Parents described feeling more in control, more self-confident, more skilled in communicating, and more able to provide security for their children. However, they also expressed a need for continuing support to maintain their improved parenting strategies.

## Introduction

Intimate partner violence (IPV) is commonly defined as psychological, physical, and/or emotional violence within an intimate, romantic relationship, and it is recognized as a serious problem with long-term effects of family members (Dutton and Kropp 2000; Sturge-Apple et al. 2012). IPV has received much attention (Borrego et al. 2008) with a large body of research showing its harmful psychological, cognitive, emotional, and social effects on both adults and children (Levendosky and Graham-Bermann 2000; Riggio 2004; Sackett and Saunders 1999) and its detrimental effect on children’s developmental trajectories (Moretti and Craig 2013). Involving children in IPV is also a form of victimization as children are physically, emotionally, and mentally unprepared to cope with such situations (Pazzagli et al. 2014). Thus they often respond to parental IPV with increased internalizing and externalizing behavioral problems (Chan and Yeung 2009). Because the direct and indirect consequences of IPV are severe for parents and children, it is crucial to find interventions to reduce the impacts. The majority of IPV interventions targeting parenting capability to date have focused solely on mothers (Borrego et al. 2008; Peled et al. 2010). Due to their often important parental role and the tendency for IPV to transmit across generations; fathers should also be involved in such interventions (Guille 2004). This study aims to evaluate an attachment-based group intervention program provided to both mothers and fathers who have experiences of IPV either as victims or perpetrators.

Most intervention programs for IPV that have been researched focus mainly on reducing parental stress and to a lesser degree on factors that contribute to problems in the parent–child relationship (e.g., Peled et al. 2010; Scott and Crooks 2007). As parent–child relationships can be affected by factors other than parental stress such as parental sensitivity and responsiveness, it is important to broaden the focus of IPV interventions to promote skills that address as many factors as possible which will contribute to improved functional parenting (Eisenberg et al. 2010). This type of all encompassing intervention would then be more effective on boosting the parent–child relationship while minimizing negative effects. Another area of research which demonstrates the importance of parent–child relationship is attachment. Attachment styles have been shown to be an IPV mediator not only in the family of origin but also subsequently in the adult family (Mauricio et al. 2007), suggesting that attachment theory should be considered when creating an effective intervention.

### Attachment and Intimate Partner Violence

According to attachment theory, the child develops attachment to the caregiver during the first year of life, regardless of the quality of the relationship (Ainsworth et al. 1978). The two most fundamental aspects of the caregiver’s role to have a secure attachment are: (1) to function both as a secure base from which the child can explore his or her surroundings; and (2) to be a safe haven to which the child can return when feeling distressed or threatened (Ainsworth et al. 1978). A secure attachment does not require perfect parenting, but it needs a caregiver who, at a minimum, responds adequately to the child’s signals. An example of responding to a child’s signals would be, if a child got upset, the caregiver would try to comfort and soothe the child rather than ignoring him/her or getting angry at him/her for expressing that emotion. The child-caregiver relationship constitutes the base from which the child shapes internal working models which function as guides on how to interpret the world, to understand others’ expectations, as well as to react to others outside the child-caregiver dyad (Cassidy and Berlin 1994). Children with a sensitive, responsive, and available caregiver will develop a secure internal working model of the world. In contrast, children with an insensitive, inconsistent, and/or unavailable caregiver will develop an insecure working model (i.e., insecure-dismissing or insecure-preoccupied; Allison et al. 2008).

There is empirical evidence that IPV affects children’s attachment. For example, Godbout, Dutton, Lussier, and Sabourin (2009) showed in their study of a large community sample that exposure to IPV is a risk factor for a child to develop an insecure internal working model. Bettman and Tucker (2011) found that the quality of attachment remains open to improvement even in late adolescence; therefore, improving parenting skills, regardless of the child’s age, are crucial to having an intervention program with an IPV focus for parents.

### Other Family of Origin Factors Influencing Parent–Child Relationships

In a literature review, Belsky (1999) showed that the most detrimental factors for the quality of the parent–child relationship were high levels of distress and depression in the parent being negatively correlated with a good relationship. In line with such results, Lavendosky and Graham-Bermann (2001) reported that several factors such as marital dissatisfaction, depression, anxiety, and stressful life events were associated with negative qualities of parenting (e.g., reduced warmth and lower degrees of child-centeredness).

Emotional regulation—the ability to control, manage, and modify positive and negative emotional responses—has been linked to attachment (Contreras et al. 2000). The child learns about regulating his/her emotions by observing how parents handle their own emotions (Cassidy and Berlin 1994; Sheffield et al. 2007). However, when both parents cannot support the child’s emotional regulation, his/her development of effective regulation skills will be impaired and thereby diminish the child’s ability to cope with difficulties in a constructive way (Cassidy and Berlin 1994). For instance, continual exposure to IPV interferes with the child’s ability to modulate arousal and to learn to self-soothe after frightening experiences. Impaired emotional regulation can also impede the child’s social functioning and emotional development (Alink et al. 2009) and is associated with poorer mental health (Eisenberg et al. 2010) and relational aggression (Conway 2005). In light of the bi-directional nature of parent–child relationships (Parke 2004), IPV may not only have a direct influence on the parent–child relationship due to reduced parenting capability, but also an indirect influence because of the child’s diminished emotion regulation making him/her more defiant and difficult to manage.

### Attachment-Based Interventions of Intimate Partner Violence

Given that attachment theory provides a framework that elucidates both parenting skills and children’s needs, attachment-based interventions provide a pertinent basis for the treatment of families with a history of IPV. Although attachment-based interventions have had promising results in improving parent–child relationships (Hoffman et al. 2006), to date few studies have used attachment-based interventions in populations affected by IPV. Research has been conducted on parent–child interaction therapy (PCIT) in which the first phase of the treatment, relationship enhancement, is based on attachment theory. In an evaluative report by Borrego et al. (2008), it was found that PCIT intervention can address issues such as the parent–child relationship in families experiencing IPV.

Another attachment-based intervention is the Circle of Security program (COS-P; Powell et al. 2013) that addresses parent–child attachment and relationship issues through focusing more broadly on both parents’ and children’s needs. The COS-P is an intervention aimed to promote attachment security by improving parents’ capacity to reflect upon the child’s mental states as well as to meet his/her emotional needs. The COS-P also takes into account three behavioral control systems that are important to a child’s development: the exploration system, the attachment system, and the caregiving system (Zanetti et al. 2011). These three systems work together toward providing a secure attachment and a safe haven while the child explores his or her surroundings. Some evidence has been found in support of COS-P interventions. For example, one study targeting women in a prison diversion program showed that the COS-P had beneficial effects by increasing mothers’ levels of sensitivity and decreasing their depression (Cassidy et al. 2010). Marvin et al. (2002) found improved parenting skills and a shift from disordered to ordered caregiving patterns in COS-P. As a result, the child’s behavior subsequently changed from an insecure pattern to a secure pattern, indicating that COS-P worked for both the parent and the child. Furthermore, two separate single-case studies (Page and Cain 2009; Pazzagli et al. 2014) using the COS-P found that the participants became more competent as parents in the management of their child’s needs and their interactions with each other.

Based on the research mentioned above and the fact that the aim of the current study was to explore, from the perspective of parents who were either perpetrators or victims of IPV, we chose to examine the attachment-based psycho-educational intervention program called, Parenting and Violence.

## Method

### Participants

The focus groups involved 26 parents (16 mothers and 10 fathers) with a median age of 38 for mothers and 44 for fathers. Almost all participants (23) were born in Sweden, one was born in another European country, and two were born in the Middle East. Most (18) were employed, four were on sick-leave, and four were unemployed. About half of all participants had three or more children, ranging in age from four months to 22 years. In total, 12 were married, 10 were single, and four cohabited with a partner.

### Procedure

Participants were recruited through a family crisis center in the west of Sweden which was offering treatment for women and men in relationships characterized by physical or psychological violence. Before being asked to participate, parents were presented with the aims and procedures of the study and written and oral assurance of confidentiality, anonymity, and the right of withdrawal at any time. After agreeing to participate, parents were provided with a consent form and the interviewer’s contact details for further information and queries about the research. The study commenced after ethical approval was granted by the Regional ethical review board in Gothenburg.

### The Intervention

Parenting and Violence is a 10-week group intervention program for parents who are either victims or perpetrators of IPV. Each group included a maximum of five participants, with mothers and fathers grouped separately. Each session was 1.5 h weekly for the duration of the program. The sessions were led by two social workers who specialized in family and network therapy and had previous experience leading IPV treatment groups. For the purpose of evaluation prior to its potential use as an on-going program, four rounds of the intervention program were carried out during 2011–2014.

The intervention was developed in connection to the evaluation project by Utväg Södra Älvsborg in collaboration with the fourth author, and is described in further detail by Persson and Dufva (2016). The main aims of the intervention were to: (1) increase parental awareness of and involvement in the child’s perception of the violence, (2) minimize the impact of violence on the child’s development, and (3) prevent the violence from following the child into adulthood and affecting his/her own future relationships. The intervention program was based mainly on attachment theory, but it was also influenced by elements of psycho-education aiming to help participants gain self-insight and strengthen their personal resources and coping skills.

The intervention consisted of ten steps that were divided into three phases of guiding questions. The first phase included three sessions that focused on the child’s emotional needs. The guiding questions for the discussions during this phase were: (a) What is the meaning of “good-enough” parenting? (b) What are the crucial emotional needs of children? and (c) What is your child like and how is he or she doing? The second phase included four sessions to enhance parents’ understanding of the attachment concept of Safe Hands, where parents focus on meeting and coping with the child’s emotional needs. The guiding questions for the discussions during this phase were: (a) What implications does the concept of “a good-enough parent” have for you? (b) What are you like in terms of parenting? and (c) What were your own parents like in terms of parenting? The third phase included three sessions that aimed at increasing parents’ awareness of the parenting skills that they needed to improve on in order to meet their child’s needs. The guiding questions for the discussions during this phase were: (a) What parenting skills of yours do you need to develop or improve? and (b) What do you need for yourself in order to develop or improve these skills?

The intention of the three phases was to allow participants to reflect upon parenting in the context of their experience with IPV. Some tools that were used were directly influenced by the COS-P intervention method. For example, the *circle of security* model was used to illustrate parent–child interactions in relation to the child’s needs to explore the world. Additionally, the parents were introduced to the *path of security* with the aid of COS-P’s “Shark Music,” illustrating how unregulated and perceived danger affects, influences, and shapes caregiving.

### Focus Groups

Approximately 1 week after the intervention program, focus groups were conducted with participants in the same groups as they had been in the intervention. The focus groups lasted approximately 60–90 min and were led by two moderators—the second author and a social worker specialized in family and network therapy.

The focus groups were used to obtain rich, in-depth information on participants’ experiences of the intervention. This approach also enabled exploration of “what, how, and why” questions and provided findings based on participants’ own opinions on the topic being researched as Smith, Harre, and Van Langenhove (1995) did. A question guide composed of open-ended questions was used to gain an understanding of how the intervention had influenced the participants’ ways of handling family situations. For example, questions addressed participants’ thoughts about the child’s situation and well-being after the intervention program. The focus groups were flexible; however, the probing questions explored participants’ experiences of the intervention in more detail than a Likert questionnaire could have done, allowing greater coverage of the research question and helping the moderators to explore and follow-up on interesting responses.

### Data Analysis

All data were analyzed using inductive thematic analysis as suggested by Braun and Clarke (2006). This method of analysis is widely used for analyzing and reporting themes within a data set. The transcripts for each group were read several times by the first author to get a genuine understanding of its content. After the second reading, coding was begun in the right margin, and notes were made highlighting discussions that were within the scope of this study. The transcripts were then reread to review the themes in the data. The themes were noted in the left margin, with the corresponding text selections highlighted; these were read again and reviewed for coding and for connections between selections and between themes. The larger themes were broken down into sub-themes. The final stage was to focus on the underlying meaning of the themes and sub-themes and select the quotes that best illustrated them. Throughout the process, the results of the various stages were discussed by the four authors to ensure that the analysis fit the data.

## Results

The analysis resulted in the themes and sub-themes presented in Table 1. These are illustrated below by quotations from focus group participants to show how the themes are grounded in the data. To give some context, we have indicated whether the quotation is from a mother or a father.

**Table 1:** Main findings from a qualitative analysis

Themes	Sub-themes
Perceived changes	Self-control
	Self-confidence
	Better communication
	Better parenting
Received support	Talking openly
	Getting insights about violence
	Recognizing more needs

### Perceived Changes

This theme describes participants’ perceptions of post-intervention changes in behavior among themselves, their partners, and their children. A recurring aspect of this theme was the parents’ descriptions of how their own improved self-control, self-esteem, and communication skills seemed to positively influence their children’s well-being and behavior.

#### Self-Control

The majority of the fathers stated that they had become calmer and better able to control themselves during conflicts and arguments. They described having learned to try to avoid violent behaviors such as shouting and losing control, as well as being less aggressive towards other family members. Some stated that the intervention had made them realize that it was not helpful nor necessary to lose control.

“There’s no point in me yelling and screaming because I only give negative energy. Can I influence the situation by getting angry? No, so you have to calm down.” (Father)

Another father explained that before attending the treatment group he used to lose control when the children were whiny or mischievous; whereas after the treatment group he was not as upset and stressed in such situations.

“I’ll deal with it in the situation if they whine, if they hit each other or something. So I will not be completely insane if someone does something stupid.” (Father)

The participants described how being calmer had influenced the children’s behavior because it had created a more relaxed and peaceful atmosphere at home.

“We have become calmer towards each other, and since we are calmer with each other, so are they [the children], too.” (Father)

#### Self-Confidence

The content in this sub-theme emerged especially in the focus groups with mothers. The mothers described having become more confident in themselves and more assertive as a result of the intervention. They also described feeling more confident about handling certain situations and not as afraid to speak up if they or their children should become victims of violence again. The following is an example of their reassurance in standing up against their perpetrators.

“Yes, I’m not afraid, as afraid of him… the children’s father, any longer.” (Mother)

Some parents described how they, prior to the intervention, had been insecure about the normality or abnormality of their children’s behaviors, and this insecurity had led them to question themselves as parents. However, the majority of the participants talked about how they had become more self-confident after sharing their concerns about and experiences of their children’s behaviors. The benefits of experiencing validation of their personal experiences from other parents during the group sessions were typically described as comforting. They also said that they no longer felt as alone and doubtful as they had before.

“The way the children react at home has made me realize that other children also react when they are away for the weekend and come home, they have to have their tantrums and… You do not feel so alone… I’ve become more confident in myself.” (Mother)

Furthermore, some mothers described feeling more secure and confident about parenting and decision-making. Talking about “good enough” parenting during the group sessions had made them realize that it was sufficient to try to do their best instead of always questioning themselves as a parent.

“I think it was pretty good to participate as I feel myself… I wasn’t so sure before if I did the right or wrong thing for my child. We all realized I think that… oh well I do not need to be perfect.” (Mother)

#### Better Communication

This sub-theme was discussed in both the fathers’ and mothers’ groups. They discussed how their ability to communicate with each other during the group sessions resulted in improved relationships with their partners and children. They also talked about how better communication with each other contributed to calmer discussions.

“Both I and the children’s father now have a better relationship, and even with the children, if I say so, it is not as much shouting and so on… but we can now have a discussion, like sit down and talk.” (Mother)

Having a better relationship since the intervention was not perceived to be restricted only to parents who were still living together.

“It feels better to have a good relationship, even though we don’t live together, and for no one to be angry toward the other.” (Father)

#### Better Parenting

The content of this sub-theme recurred in many parts of the focus group discussions and could reflect the attachment concepts of “safe hands” and “good enough parenting” used in the intervention. The fathers described having increased accountability for their actions, and they emphasized the importance of providing emotional support according to the child’s own needs.

“When you meet them, you have to meet them with the feelings they have and confirm that feeling they come with.” (Father)

Participants explained how scared they had felt prior to the intervention about letting their children explore their surroundings, and they described an improvement in that they had acquired the ability to attend to their children’s needs while letting them explore their surroundings and supporting their explorations.

“Now my children are allowed to go to the store by themselves and buy ice cream and it works really well.” (Mother)

The majority described their previously family environment as having been full of shouting and violence but said that it had become calmer after the intervention. They also described having gained the capacity to explain rules and to set limits.

“We talked and like, you can’t do that… shouting and hitting, it doesn’t matter how angry you are at Mom, you can’t kick and hit Mom.” (Father)

Setting rules included teaching their children how to be more helpful at home. The parents talked about nagging and shouting less when they wanted their children to help in the home.

“He [the father] has talked to the children about cleaning their room every day and making the beds and making sure they stay organized.” (Mother)

In this theme, parents described behavioral changes in themselves and in their children as a result of their participation in the intervention, which helped them to become calmer and to avoid aggressive behaviors. Furthermore, after participating in the intervention, they described how they had developed the confidence in that they had learned how to handle violent situations; they became more aware of their children’s behaviors, and they gained confidence in their parental capabilities.

### Received Support

This theme reflects descriptions of the impacts of the intervention in relation to the support they had received and the knowledge they had acquired. Foremost, the participants stressed the importance of being able to speak openly about their problems. Many also said that it was not until they had attended the group sessions that they had learned what counts as violence and realized that they themselves were victims.

#### Talking Openly

Participants felt particularly confident and comfortable in sharing their stories and problems with others because they all had experienced IPV. Both the mothers and fathers reported that the intervention had helped them talk about their problems and worries with each other, which were otherwise difficult to talk to about more openly in the group.

“It is a personal issue and it is something you’d rather not share with your colleagues or teammates… here is more like… here we can talk about our issues and worries.” (Father)

Furthermore, participation in the intervention had given the participants the opportunity to talk about their concerns and problems without being judged and to receive confirmation that they were not alone in experiencing violence.

“To talk and such, as well as dare to say exactly everything you want to say, because you know that the others have experienced the same situations.” (Mother)

#### Insights About Violence

In the focus groups, the mothers in particular described having received help during the intervention to realize that they had been victims of violence. They shared their problems with each other and received more information about violence.

“Had I not gotten in touch with the center here, I wouldn’t have gone to this group because I didn’t see myself as being abused or anything.” (Mother)

The mothers’ group explained that, before the intervention, only physical abuse counted as violence to them. After participation, they learned that violence could mean suffering more than physical abuse; it could include psychological abuse or the loss of their privacy.

“Yes, I see a lot clearer now what happens and what I encounter and now, I’ve got a different way of thinking.” (Mother)

Participants explained that taking part in the intervention had been a support and reassurance for them as they received information about where to seek help if they ever again become a victim of violence.

“It is very reassuring to have someone that knows a little bit about violence and know where to go when help is needed.” (Mother)

#### Recognizing More Needs

Despite the positive attitudes the participants had towards the intervention, they expressed fears of forgetting what they had learned and also the fear of relapsing into previous behaviors.

“We have gotten close to each other and that is nice. It is just that you can’t forget it after the treatment group is over. That is what I’m afraid of, that I’ll end up going back to my old behavior.” (Father)

The majority said that they wished the intervention could continue for a longer period:

“I would have liked it to be another 10 weeks.” (Mother)

The participants also stated that they wished their children could receive information about violence at an early age, just as children get information in school about drugs, for example.

“The school should talk about violence… it is the natural way of learning that in school, too.” (Mother)

This theme highlighted the support the participants received from taking part in the intervention. They saw the intervention as informative about what violence is and where to get help if needed. Since the topic of IPV is commonly seen as taboo in Swedish society, participants described the group sessions as helpful in the sense that it gave them an opportunity to disclose and talk about their experiences with other parents who had experienced similar problems. However, the participants expressed concerns about possible relapse to their previous behaviors and they expressed a need for further support.

## Discussion

The findings from this study offer insights into the experiences described by victims and perpetrators of IPV who participated in the Parenting and Violence intervention focused on parent–child relationships. Parents described themselves as being more in control, more self-confident, better skilled at communicating, and better able to provide security for their children after the intervention.

Both mothers and fathers reported that they had improved their impulse-control and became more attentive to their children’s needs, which they believed had resulted in themselves being calmer and in better control in conflict situations. These findings are in line with research showing that parents’ emotion regulation plays an important role in parent–child attachment (Obsuth et al. 2014). In the present study, the parents also talked about themselves as being more able to respond to their children’s behaviors with self-confidence (for the mothers) and self-control (for the fathers). Both of these factors have been recognized as restraining over-reactive parental discipline (Kohlhoff and Barnett 2013; Lesniowska et al. 2015).

The parents described how the Parenting and Violence intervention had improved their capacity to communicate both with their children and with their partners. According to Pazzagli et al. (2014), partner relationships are likely to improve with increased communication skills. Therefore, our finding indicates that parents who participated in the groups might have improved their partner relationships as well. Because most previous attachment-based intervention studies do not target families with a history of IPV, this study provides important information about the potential effects that an attachment-based intervention such as Parenting and Violence can have on communication skills in these families.

After the intervention, parents expressed that they had a sense of being good enough as parents. This perception of their parental capability became apparent to themselves as they shared their concerns with the other parents during the groups. For mothers, good enough parenting was expressed in terms of believing in themselves and believing that what they were doing for their children was right; whereas for fathers, good enough parenting meant offering their children increased emotional support. According to attachment theory, the parental role of being wiser and stronger makes it possible for children to confide in their parents and seek help from them (Marvin et al. 2002). The parents in this study expressed feeling that they had become better at making decisions and setting rules and limits which could lead to healthier, more balanced children and not perpetuate negative characteristics into their adult life. For example, research indicates that children whose parents demand positive behavior in a responsive manner and set behavior limits in the child’s interest are rated higher on social and school adjustment (Chen et al. 1997; Jutengren and Palmérus 2007).

The parents described how discussing parenting issues with others in a group was especially helpful in facilitating their recognition of negative parenting practices, and how this, in turn, resulted in their adopting new, more functional, strategies such as reasoning about boundaries in response to child transgressions. In the parents’ discussions in the focus groups, the intervention was also perceived as therapeutic and informative because it helped them both to talk openly and to get more information about violence. Open discussions helped participants realize that there were others experiencing IPV with whom they could share their concerns and learn to deal better with violent situations. Learning more about IPV helped mothers to realize that they had been victims of violence. New insights about being a perpetrator were not described in a direct way; however, fathers’ expressions of fear about relapsing into previous behaviors could reflect a sense of understanding of their aggressive and violent behaviors. This shows that families with a history of IPV need not only help in the field of parent–child attachment but also therapeutic support of others who have experienced IPV in order to gain a sense of understanding of their own violence. The findings from the focus groups show that the parents seemed to learn from discussions over the course of the intervention and apply that learning to their individual families. By having their experiences validated through others, parents got insight into their own situation and described that they were less inclined to accept IPV and more inclined to seek help whenever it occurred.

It is important to note that many of the parents expressed a need for continuing support to maintain their improved parenting strategies. These needs could be met by extending the duration of the program and offering support over a longer, and perhaps less intense, period of time. Another concern is that the materials in the intervention were in English although the participants all had Swedish as their dominant language. Although not mentioned in the findings, it was expressed in the focus groups that the educative material would have been easier to understand and integrate if it had been given in Swedish.

This study has limitations that are important to address when interpreting the results. First, it should be acknowledged that the participating parents had self-selected to take part in the treatment intervention which could indicate a difference between those who participated and those who did not. In addition, the majority of participants were living in the same home and were willing to work at co-parenting. As a consequence, participants seemed highly motivated and aware of their own parental shortcomings. Due to this selection bias, the results should not be considered representative of parents who participate in treatment interventions involuntarily. Second, as is normally the case when using retrospective qualitative data, the statements made by the participants are likely to be subjected to confounding factors such as memory distortion and a desire to meet the researchers’ expectations. However, it is also relevant to point out that those who conducted the focus groups were not the same people who carried out the intervention, which may have contributed to a more open and honest discussion. Lastly, this study focused only on the parents’ perspectives; future studies would benefit from interviewing the children to determine if the new techniques taught in the intervention were implemented and as effective as the parents’ perception. Interviewing the children would also provide information about the child’s perceptions of their parents’ capabilities to meet their needs after their parents had participated in a similar intervention.

In sum, this study has evaluated an attachment-based intervention program specifically designed to treat both victims and perpetrators of IPV with both mothers and fathers as participants. According to the participants’ reports, they experienced the intervention as supportive and confirming of their role as parents, describing themselves as feeling more in control, more self-confident, more skilled in communicating, and more able to provide security as well as a physically and psychologically healthier environment for their children. Although they also expressed a need for continuing support to maintain their improved parenting strategies, the fundamental approach to the treatment of IPV used in the parenting and violence program seems worthy of further evaluation and development.

## Conclusion

Although the Parenting and Violence intervention program has yet to be evaluated in effect studies, participating parents report that their parenting skills improved in a number of important ways. Despite focusing on the parent-child relationship, this intervention program also seems to benefit competencies pertaining to inter-partner relationships. In addition, the possible awkwardness of exposing private experiences in a group-treatment situation (cf. Strand et al. 2015) does not seem to have been an issue. In light of the combination of the results of this study and a well-founded theoretical basis, the Parenting and Violence intervention program brings promise of a treatment that can improve the life circumstances for both victims and perpetrators of IPV, as well as for their children.
